# 复发性小细胞肺癌治疗进展

**DOI:** 10.3779/j.issn.1009-3419.2017.03.08

**Published:** 2017-03-20

**Authors:** 斌 刘, 建文 秦, 静敏 周

**Affiliations:** 300222 天津，天津市胸科医院呼吸与危重症科二病区 Department of Respiratory and Critical Care, Tianjin Chest Hospital, Tianjin 300222, China

**Keywords:** 肺肿瘤, 复发, 化疗, 靶向治疗, Lung neoplasms, Relapsed, Chemotherapy, Targeted therapy

## Abstract

小细胞肺癌（small cell lung cancer, SCLC）是一种高度恶性肿瘤，初始治疗对放化疗均较敏感，缓解率高，但治疗后易发生耐药并复发。虽然近年来肺癌的治疗发生了翻天覆地的变化，但SCLC复发后的治疗依然是临床上棘手的难题。鉴于复发性SCLC对现有化疗药物的耐药性严重，国内外对复发性SCLC的研究多集中在新药物研制、化疗方案优化及靶点药物的开发等临床试验上。本文对近年来复发性SCLC的化疗和精准治疗的各项研究和文献报道进行总结与评价，期望能够对复发性SCLC的临床治疗有所启发，并为复发性SCLC治疗的临床研究指引方向。

## Abstract (en)

Small cell lung cancer (SCLC) is a highly malignant tumor. The initial treatment of radiotherapy and chemotherapy are more sensitive, high remission rate, but susceptible to drug resistance and relapse after treatment. Although the treatment of lung cancer has undergone enormous changes in recent years, treatment for relapsed SCLC is still a difficult problem in clinical field. In view of the serious resistance of recurrent SCLC to the existing chemotherapeutic drugs, the research on recurrent SCLC around the world is focused on the clinical trial of new drug development, optimization of chemotherapy regimen and target drug development. This paper summarize and estimate studies and literature reports of chemotherapy and precision therapy for relapsed SCLC, hopefully it could help clinicians treat relapsed SCLC and give us clinical research direction for relapsed SCLC in the future.

小细胞肺癌（small cell lung cancer, SCLC）是一种神经内分泌肿瘤，约占肺癌总数的15%^[1]^，由于其较强的侵袭性和早期易发生血行转移及淋巴转移，预后极差。仅有不足5%的临床分期为Ⅰ期^[2]^的（T1-2N0M0）患者能够从手术中获益。其余患者一线经放化疗治疗后能取得较高的客观缓解率（objective response rate, ORR），但大约80%局限期SCLC患者和几乎所有广泛期SCLC患者均会在1年内复发。以往认为复发后SCLC患者有效治疗药物较少，预后差。近年来随着精准医疗的发展、免疫靶向药物及新型化疗药物的研制以及诊疗技术和诊疗水平的不断提高，SCLC的治疗方法逐渐增多，我们从这些新的研究进展中看到了SCLC治疗的希望。

## 1 复发性SCLC的治疗现状

SCLC是对化疗高度敏感的恶性肿瘤，有效率高达80%左右，但非常遗憾的是绝大多数患者均会复发。为延长SCLC复发时间有些医生采取维持治疗的策略，但有研究^[3]^表明无论是给予化疗还是干扰素或生物制剂的维持治疗，接受维持治疗的SCLC患者总生存期（overall survival, OS）都没有明显延长，仅产生微弱的临床获益。临床上将复发SCLC患者分为3类^[4, 5]^：①难治性复发：一线化疗过程中疾病进展；②耐药复发：一线化疗结束后3个月内疾病进展；③敏感复发：一线化疗结束3个月以后疾病进展。现有指南和专家共识^[5]^推荐可以依据复发时间选择二线治疗，3个月内疾病复发进展的患者推荐参加临床试验。3个月-6个月内复发者推荐拓扑替康、伊立替康、吉西他滨或紫杉醇治疗。6个月后复发可选择应用初始化疗方案。

对复发的SCLC多药联合是否有效呢？考虑到SCLC一线化疗是以铂类为基础的联合化疗，SCLC复发后对铂类耐药率高，因此二线治疗并不常规联合铂类。对于复发性SCLC目前美国国立综合癌症网络（National Comprehensive Cancer Network, NCCN）2016年第1版指南的可选方案中CAV（环磷酰胺、多柔比星、长春新碱）是唯一联合化疗方案，但早有研究^[6]^证实CAV方案无论从疗效还是不良反应方面均逊于单药拓扑替康。另一口服三药（洛莫司汀、环磷酰胺、依托泊苷）联合方案治疗复发性SCLC ^[7]^和静脉CAV方案对比，结果提示虽然该方案可行性高但并不优于静脉CAV化疗。既往的吉西他滨联合伊立替康^[8]^的Ⅱ期临床实验（包括10例难治性复发，20例敏感复发）提示其ORR为36.7%，中位OS为14.4个月，1年生存率为51%，尚无后续报道，且较拓扑替康仍未能显现出明显优势。可见多药联合化疗对复发性SCLC报道并不少，但能够带来的生存获益的方案较少且大多被巨大的毒副作用所抵消。因此近十年来对于SCLC的研究重点大多放在新药（如拓扑异构酶抑制剂、蒽环类、烷化剂和铂类等）的研究上。

到目前为止拓扑替康仍是唯一被食品药品监督管理局（Food and Drug Administration, FDA）批准用于复发型SCLC的二线治疗药物，Horita等^[9]^评价含拓扑替康方案对复发性SCLC的作用，对14篇文章共1, 347例患者进行*meta*分析，其中难治性复发患者6个月OS率为37%，1年OS率为9%，ORR：5%。敏感复发6个月OS率为57%，1年OS率为27%，ORR：17%。不良反应方面3级/4级中性粒细胞减少69%，3级/4级血小板减少41%，3级/4级贫血24%，化疗相关死亡2%。可见拓扑替康对于敏感复发SCLC效果尚可而对于难治性、耐药复发的SCLC患者预后差并且不良事件发生率高，基于这样的治疗现状复发性SCLC的治疗急需取得突破性的进展。

## 2 复发性SCLC的化疗进展

### 2.1 敏感复发的SCLC化疗进展

2016年*Lancet*杂志公布了PEI方案节拍化疗（每周顺铂25 mg/m^2^，隔周依托泊苷60 mg/m^2^，隔周伊立替康90 mg/m^2^）治疗敏感复发SCLC患者的随机Ⅲ期研究^[10]^，结果显示PFS和OS显著长于单药拓扑替康组（5.7个月 *vs* 3.6个月，18.2个月 *vs* 12.5个月，*P*=0.007, 9），但与拓扑替康相比不良事件发生率也相应增加，中性粒细胞减少（83% *vs* 86%）、贫血（84% *vs* 28%）、白细胞减少（80% *vs* 51%）、3级/4级粒细胞缺乏伴发热（31% *vs* 7%）、3级/4级血小板减少（41% *vs* 28%）、严重不良事件（4% *vs* 10%）。这项研究为敏感复发SCLC的二线治疗指明了新的方向，同时因为节拍化疗带来的不良反应也对临床医生调节药物剂量、用药时间及辅助支持治疗的策略提出了挑战。另外Morise等^[11]^对低剂量伊立替康（60 mg/m^2^，d1、d8和d15，28 d为1个周期）二线单药治疗SCLC的疗效和安全性进行了一项回顾性研究，ORR为32%，无进展生存期（progression free survival, PFS）和OS分别为2.9个月和5.3个月，3级/4级粒细胞减低，腹泻和恶心/呕吐的发生率分别为21%、4%和5%，证实了伊立替康对于老年复发患者有效性和安全性。氨柔比星在日本也被批准用于复发性SCLC的治疗^[12]^。罗秋平等^[13]^的*meta*分析证实了氨柔比星对复发SCLC的ORR较高达到了46%-51%优于拓扑替康且不良反应低于拓扑替康，其中位OS达到了9.2个月-10.3个月与拓扑替康相近。尚有研究^[14]^证实氨柔比星对中枢神经系统转移有效。虽然关于氨柔比星临床试验多来源于日本，但是Horita等^[15]^*meta*分析也发现氨柔比星对敏感复发的SCLC欧美患者也有效，ORR、疾病控制率（disease control rate, DCR）分别为43%和69%均优于拓扑替康，该药在不同国家、种族的疗效有待进一步临床观察。贝洛替康在复发性SCLC的临床研究规模均较小，其中一项50例单药贝洛替康治疗复发性SCLC的研究^[16]^提示敏感复发SCLC患者ORR为20%，OS为6.5个月，PFS为2.8个月；韩国的一项Ⅱ期临床研究^[17]^提示贝洛替康二线治疗SCLC的缓解率（response rate, RR）达到了24%，中位OS为9.9个月，但无后续的报道。替莫唑胺是一种口服烷化剂，其疗效与O^6^-甲基鸟嘌呤甲基转移酶（O^6^-methylguanine DNA methyltransferase, MGMT）基因启动子的甲基化相关^[18, 19]^，该药单独用于复发性SCLC的一项的Ⅱ期临床研究^[20]^表明口服替莫唑胺不良反应少，总的ORR仅为20%，对于脑转移患者口服该药不失为一种可选治疗方法。紫杉醇也曾广泛用于SCLC二线化疗，紫杉醇联合卡铂^[21]^，联合多柔比星以及联合吉西他滨^[22]^等都有一些报道，虽然取得了较好的DCR但并未在生存获益上取得相应的优势，而且较高的毒性也伴随而来。吡铂和洛铂是新型铂化合物，其神经毒性和肾毒性更小，但对复发性SCLC未能显示出生存优势^[23, 24]^，有待进一步对新型铂类开展优化临床合理用药的研究。

总之，对于敏感复发的SCLC患者只有PEI方案节拍化疗较拓扑替康有明显的生存获益，除此以外氨柔比星的二线治疗地位也逐渐确立，也许PEI方案的优化是才是未来的联合化疗的研究重点，另外我们也期待着氨柔比星、贝洛替康等新药在SCLC的进一步研究。

### 2.2 难治、耐药复发的SCLC化疗进展

难治复发和耐药复发的SCLC患者生存时间短，对化疗药物的RR＜10%，OS＜6个月^[25]^。可喜的是近年来有多项研究证实了氨柔比星在此领域的有效性和安全性。其中氨柔比星和拓扑替康随机对照的Ⅲ期临床研究^[26]^表明二者中位OS相近（7.5个月 *vs* 7.8个月），但对于难治复发SCLC氨柔比星明显优于拓扑替康：中位OS、中位PFS和ORR（6.2个月 *vs* 5.7个月，4.1个月 *vs* 3.5个月，31.1% *vs* 16.9%，均*P*＜0.05），3级以上不良事件发生率氨柔比星组更少。Horita等^[15]^*meta*分析发现氨柔比星对难治性复发SCLC患者其ORR、DCR、1年OS率分别为19%、68%和19%均较拓扑替康增加，不良反应发生率和拓扑替康相似；而氨柔比星（每日35 mg/m^2^，第3-5天）联合拓扑替康（每日0.75 mg/m^2^，第1-5天）治疗耐药复发SCLC患者ORR达27%^[27]^。另有氨柔比星联合卡铂对难治性复发性SCLC的研究^[28]^结果显示，ORR为34%，中位PFS为3.5个月，中位OS为7.3个月，最主要的Ⅲ度/Ⅳ度毒性反应为中性粒细胞减少（79%），非血液系统毒性反应大多不严重，无治疗相关性死亡发生。此外2014年的一项Ⅱ期临床研究^[29]^首次证实了苯达莫司汀对耐药复发的SCLC的有效性，其中位疾病进展时间（time to progression, TTP）为3.4个月，RR为17%，常见的3级/4级的不良事件发生率较低，NCCN（2016年第1版）指南也推荐该药为复发SCLC的可选治疗。纳米蛋白结合型紫杉醇无需使用有毒溶剂，大幅度提高了紫杉醇的用量和紫杉醇在肿瘤组织的分布，近期日本京都大学医学院的一项回顾性分析^[30]^提示纳米蛋白结合紫杉醇（第1、8、15天给予100 mg/m^2^，每28天1个周期）在9例难治、耐药复发的SCLC患者治疗中ORR达到了33%并且毒性轻微，而这3例患者中2例患者是在传统的溶剂型紫杉醇治疗后复发，初步显示了增加紫杉醇在癌组织分布后的治疗效果。Jacot等^[31]^观察表柔比星联合异环磷酰胺对复发性SCLC化疗，中位OS仅为3.9个月，但单因素分析发现在此之前未用蒽环类药物患者中，表柔比星联合异环磷酰胺有更好疗效，提示在依托泊苷与顺铂标准方案治疗后复发的SCLC患者可试用此方案。

归纳上述各项方案不难发现氨柔比星及其联合方案对于难治和耐药复发的SCLC显示出较好的前景，或能取代拓扑替康成为耐药和难治复发SCLC的首选化疗药物。另外苯达莫司汀、蛋白结合型紫杉醇以及传统的表柔比星联合异环磷酰胺等药物也在不同方面取得了一定的优势，但仍有待于进一步研究。

## 3 SCLC的精准治疗

### 3.1 SCLC驱动基因的研究进展

SCLC是肺神经内分泌肿瘤，*TP53*基因、*RB1*基因、Notch家族基因和MYC通路等是近年来SCLC的研究热点，George等^[32]^对110例SCLC的全基因组测序发现几乎所有的SCLC均会出现抑癌基因*TP53*和*RB1*的失活，25%的SCLC出现Notch家族基因出现失活突变，并且在小鼠的SCLC模型上Notch通路激活能够大大减少肿瘤细胞的数量，使鼠的生存期延长。上海胸科医院的姜丽岩等^[33]^的全基因组测序也发现抑癌基因*TP53*和*RB1*的失活以及缺失突变等与SCLC的发生发展密切相关，同时发现SRSF1是丝氨酸/精氨酸的剪接因子，SRSF1的DNA的拷贝数增加以及mRNA的过表达与SCLC的预后不良相关，SRSF1与SCLC的发生密切相关，同时在DNA的修复和肿瘤化疗的敏感性方面起关键作用，SRSF1可作为SCLC的预后标志物为SCLC的个体化治疗提供理论基础。SCLC驱动基因的研究仍在继续，我们也终将揭开SCLC靶向治疗的面纱。

### 3.2 抗体藕联药物

在神经内分泌肿瘤中Notch信号发挥抑制肿瘤的作用，DLL3是Notch信号的配体，它能够使Notch和DLL1（Notch的另一配体）重新定位或者保留在晚期溶酶体或者高尔基体，抑制他们定位在细胞表面，从而抑制Notch通路的信号传递。DLL3的蛋白表达分析发现，正常肺组织和肺鳞癌没有DLL3的表达，未治疗的SCLC有72%表达，复发耐药的SCLC有85%表达，且在SCLC细胞表面表达增加。更重要的是DLL3表达在肿瘤起始细胞和耐药肿瘤细胞，因此理论上如果能够靶向DLL3就可以根除肿瘤复发和耐药。Rovalpituzumab tesirine（Rova-T）是DLL3的靶向性单克隆抗体和细胞毒药物的耦联物^[34]^。2015年世界肺癌大会（World Conference on Lung Cancer, WCLC）首次报道了Rova-T对可评价的60例复发性SCLC患者的Ⅰ期临床研究^[35]^，结果提示总ORR为20%，总的临床获益率（clinical benefit rate, CBR）为75%，其中28例DLL3阳性的患者中ORR：39%，CBR：75%。2016年美国临床肿瘤学会年会（American Society of Clinical Oncology, ASCO）年会该研究更新的结果显示中位OS：4.6个月，1年OS：18%；在DLL3≥50%（DLL3hi）的26例患者中，中位OS为5.8个月，1年OS为32%，CBR：89%；12例DLL3hi的三线治疗患者ORR为50%，CBR为92%，远远高于传统化疗药。Rova-T不但疗效好且毒性低，3级以上药物相关不良事件：浆膜腔积液11%，血小板减少12%，皮肤反应8%，由此可见该药对于复发耐药的且体能较差的SCLC患者更具优势。期待着Rova-T的Ⅱ期实验能够得到更加鼓舞人心的结果。

Trop-2是一种细胞表面跨膜糖蛋白受体和该信号转导子，在正常组织表达很低，肿瘤组织则出现过表达。Sacituzumab govitecan（IMMU-132）^[36]^是Trop-2的单克隆抗体和伊立替康的活性代谢产物SN-38组成的抗体耦联物，IMMU-132在复发性SCLC的治疗中也获得了较好的ORR，此外SC-002是另一种神经内分泌肿瘤干细胞的抗体耦联药物，他在复发性SCLC的1a期/1b期临床研究（NCT02500914）的结果也值得期待。

### 3.3 免疫检查点抑制剂

SCLC基因组不稳定，存在较高突变负荷（TMB），理论上对免疫检查点治疗更敏感。目前应用最广泛的免疫检查点抑制剂包括CTLA-4、PD-1及其配体PD-L1的抑制剂。

Ipilimumab是CTLA-4抑制剂，用于SCLC的治疗曾被寄予厚望，非常遗憾该药在一线联合化疗治疗SCLC的Ⅲ期临床研究结果是阴性的^[37]^，分析原因为：①Ipilimumab联合化疗没能引起肿瘤微环境中T细胞的活化，因此没有激起足够的抗肿瘤免疫。②化疗抑制了T细胞的活化增殖。Pembrolizumab是PD-1单克隆抗体，KEYNOTE-028研究发现在20例PD-L1阳性的化疗失败的广泛期SCLC的7例有效^[38]^，其中6例患者处于持续的治疗中，ORR高达35%，提示该药在PD-L1高表达的SCLC中强劲持久的疗效。其他关于Pembrolizumab的研究包括NCT02359019（标准化疗后Pembrolizumab维持治疗的Ⅱ期研究）和NCT02403920（Pembrozulimab+放疗联合化疗的Ⅰ期研究）均在进行中。

CTLA-4的主要作用在T细胞活化早期的激活阶段，而PD-1/PD-L1主要作用在外周或肿瘤组织中T细胞活化晚期的效应阶段，仅阻断其中的一条途径，自然会导致其他途径的活化，二者的联合阻断才可能是有希望的治疗策略^[39]^。2016年ASCO公布了CheckMate032的研究^[40]^结果提示Ipilimumab 3 mg/kg联合Nivolumab（PD-1的另一单抗）1 mg/kg每3周1个周期的方案治疗复发性SCLC取得了较好的疗效，其中位OS：7.7个月，1年生存率：42%，ORR：23%较单药拓扑替康治疗复发性SCLC更优，并探索性分析发现PD-L1表达情况与疗效并不相关，该研究认为对于耐药复发SCLC联合治疗具有持久的抗肿瘤活性，生存结果令人鼓舞。此外Nivolumab与单药（拓扑替康或氨柔比星）化疗对复发耐药SCLC的研究（CheckMate 331研究）也正在进行中。Atezolizumab是PD-L1的抗体，其作用也是阻断T细胞表面PD-1与PD-L1的相互作用，以重新激活抗肿瘤免疫，对于SCLC的治疗也在研究中。

上述研究可以看出免疫检查点抑制剂在SCLC治疗尚处于起步阶段仍需进行更多的探索，PD-L1的高表达、肿瘤的突变负荷等是否能成为免疫检查点抑制剂的预测因子还有待进一步验证。

### 3.4 Aurora激酶A抑制剂Alisertib

Aurora激酶家族（A, B, C）为丝/苏氨酸蛋白激酶，在有丝分裂的染色体排列、分离和胞质分裂中起重要作用。Aurora激酶A是该家族的重要成员，对中心体的成熟和纺锤体的装配起重要作用，与人类多种恶性肿瘤相关。Alisertib是口服选择性aurora激酶A抑制剂，对多种实体肿瘤有效，Melichar等^[41]^研究表明该药在SCLC难治性复发RR高达25%，敏感复发RR为19%，与拓扑替康相当，但尚未明确Alisertib的靶基因。2016年欧洲临床肿瘤学会年会（European Society for Medical Oncology, EMSO）会议上Siraj Ali报道了他们采用杂交捕获的全基因组测序检测了689例SCLC的基因组变异，同时对一些SCLC中已知的基因变异进行靶向测序，发现7例患者有*MYCL1*基因融合。其中有1例非吸烟*MYCL1*融合的SCLC患者，接受了Alisertib治疗并接受了9个月的Niovlumab治疗，疗效接近CR，PFS长达18个月。从中我们得到启发，*MYCL1*扩增在SCLC的发生率较高，Alisertib或许能用于*MYCL1*扩增的患者，如果后续得到大样本的验证，那么SCLC治疗就真正走向了精准医学。

### 3.5 PARP通路抑制剂

PARP是一种DNA修复酶，在DNA修复与细胞凋亡中起重要作用，Veliparib是一种新型高选择PARP1抑制剂，通过干扰细胞DNA修复过程而起作用，使肿瘤对损伤DNA的化疗药物变得更加敏感，Veliparib联合标准化疗初治广泛期SCLC的抗肿瘤作用已被证实^[42]^。2016年ASCO报道的一项Veliparib联合替莫唑胺治疗复发性SCLC的2期临床研究取得了8.2个月的中位OS，RR为39%较单药替莫唑胺改善显著，该研究也提示SLFN11有可能是预测Veliparib等PARP抑制剂疗效的生物标志物^[43]^。目前发现的PARP的抑制剂还有Talazoparib、olaparib等药物，它们对于SCLC的治疗也都处于探索中。

## 4 SCLC的抗血管生成治疗

贝伐珠单抗是血管内皮生长因子（vascular endothelial growth factor, VEGF）的单克隆抗体，它在SCLC的抗血管生成治疗中的研究最多，涉及一线、二线和维持治疗。目前规模最大一项Ⅲ期研究^[44]^显示贝伐珠单抗联合EP方案（足叶乙甙+顺铂）一线治疗广泛期SCLC提示PFS、OS的均延长，但没有统计学改善，联合治疗毒性可接受。希腊的一项伊立替康联合贝伐珠单抗治疗28例耐药复发SCLC的Ⅱ期研究^[45]^提示ORR：25%，中位PFS：3.2个月，中位OS：6.3个月，3级/4级不良反应中性粒细胞减少：7.1%，蛋白尿3.5%，与历史对照毒性低疗效好，应对此方案进一步临床研究。另一项拓扑替康联合贝伐珠单抗治疗50例复发性SCLC的Ⅱ期研究^[46]^结果与之类似，与历史对照3个月的PFS分别为65%和50%，虽有所改善但未达到预期目标，总体OS：7.4个月，中位PFS敏感复发组（27例）和耐药复发组（23例）分别为6.24个月和2.91个月，其3级/4级/5级不良反应78%，因此应慎重选用该法方案。

阿柏西普（Aflibercept）与VEGFR-1和VEGFR-2有很高的亲和力，在与拓扑替康联合治疗铂类耐药的SCLC患者中3个月PFS有所改善^[47]^，但OS并没有明显改善且不良反应相应增加。此外舒尼替尼、索拉非尼、帕唑替尼等抗血管生成药物的临床试验提示患者未获得明显受益且不良反应增加。总之目前报道的复发性SCLC的抗血管生成治疗较多但临床明显获益的很少，需要进一步临床试验寻求突破。

## 5 小结

复发SCLC二线化疗进展缓慢，但PEI方案节拍化疗、氨柔比星、贝洛替康等药物取得了一些令人可喜进展。精准医疗的发展打破了SCLC领域的沉寂，基因分析是目前实现精准医疗非常重要的部分，近年来发现的*TP53*基因、*RB1*基因以及Notch、Myc信号通路可能是未来SCLC精准医疗的研究方向。抗体耦联药物Rova-T等在SCLC的研究中大放异彩，成为精准医疗行列中的新生力量。免疫检查点抑制剂在SCLC治疗的研究仍然势头强劲，并且中国也已经启动免疫治疗在SCLC二线中的研究，这些研究让SCLC的精准治疗充满了挑战和希望。
